# Multisensory Audiovisual Processing in Children With a Sensory Processing Disorder (II): Speech Integration Under Noisy Environmental Conditions

**DOI:** 10.3389/fnint.2020.00039

**Published:** 2020-07-14

**Authors:** John J. Foxe, Victor A. Del Bene, Lars A. Ross, Elizabeth M. Ridgway, Ana A. Francisco, Sophie Molholm

**Affiliations:** ^1^The Cognitive Neurophysiology Laboratory, Department of Neuroscience, The Ernest J. Del Monte Institute for Neuroscience, University of Rochester School of Medicine and Dentistry, Rochester, NY, United States; ^2^The Cognitive Neurophysiology Laboratory, Department of Pediatrics, Albert Einstein College of Medicine and Montefiore Medical Center, Bronx, NY, United States; ^3^The Dominic P. Purpura Department of Neuroscience, Albert Einstein College of Medicine, Bronx, NY, United States

**Keywords:** cross-modal, audiovisual, autism spectrum disorders, multisensory integration, ASD, sensory integration, SPD

## Abstract

**Background**: There exists a cohort of children and adults who exhibit an inordinately high degree of discomfort when experiencing what would be considered moderate and manageable levels of sensory input. That is, they show over-responsivity in the face of entirely typical sound, light, touch, taste, or smell inputs, and this occurs to such an extent that it interferes with their daily functioning and reaches clinical levels of dysfunction. What marks these individuals apart is that this sensory processing disorder (SPD) is observed in the absence of other symptom clusters that would result in a diagnosis of Autism, ADHD, or other neurodevelopmental disorders more typically associated with sensory processing difficulties. One major theory forwarded to account for these SPDs posits a deficit in multisensory integration, such that the various sensory inputs are not appropriately integrated into the central nervous system, leading to an overwhelming sensory-perceptual environment, and in turn to the sensory-defensive phenotype observed in these individuals.

**Methods**: We tested whether children (6–16 years) with an over-responsive SPD phenotype (*N* = 12) integrated multisensory speech differently from age-matched typically-developing controls (TD: *N* = 12). Participants identified monosyllabic words while background noise level and sensory modality (auditory-alone, visual-alone, audiovisual) were varied in pseudorandom order. Improved word identification when speech was both seen and heard compared to when it was simply heard served to index multisensory speech integration.

**Results**: School-aged children with an SPD show a deficit in the ability to benefit from the combination of both seen and heard speech inputs under noisy environmental conditions, suggesting that these children do not benefit from multisensory integrative processing to the same extent as their typically developing peers. In contrast, auditory-alone performance did not differ between the groups, signifying that this multisensory deficit is not simply due to impaired processing of auditory speech.

**Conclusions**: Children with an over-responsive SPD show a substantial reduction in their ability to benefit from complementary audiovisual speech, to enhance speech perception in a noisy environment. This has clear implications for performance in the classroom and other learning environments. Impaired multisensory integration may contribute to sensory over-reactivity that is the definitional of SPD.

## Introduction

Sensory Processing Disorder (SPD) is characterized by hypo- or hypersensitivities to sensory inputs that cause significant disruption to everyday activities (Miller et al., 2009; Schoen et al., 2009). At its core, SPD represents a failure to appropriately modulate the effects of incoming sensory inputs, and in turn, this raises the issue of whether the integration of inputs across sensory systems is functioning appropriately in this population. The principal function of the multisensory integration system is to combine the signals that enter the brain through the separate sensory epithelia so that the different forms of energy emanating from the same object or event will be treated as a unified percept. In other words, the multisensory system solves the binding problem, and in doing so, it serves to simplify the world and leads to substantial improvements in behavioral efficiency (Molholm et al., 2002; Foxe and Schroeder, 2005; Rowland et al., 2007; Senkowski et al., 2007; Gingras et al., 2009; Mahoney et al., 2015; Shaw et al., 2020). By unifying segregated sensory events, the multisensory system also serves to unclutter the perceptual landscape. Consider the alternative, where the various sensory inputs might be perceived as separate events because of a failure of sensory integration. One might well expect that this would lead to a general inundation of central processing capacities, and perhaps an obvious outcome would be a general sensory defensiveness or over-responsivity.

While sensory processing irregularities are often associated with canonical neurodevelopmental disorders, especially Autism Spectrum Disorder (ASD) and Attention Deficit Hyperactivity Disorder (ADHD), there is no necessary reason that one should expect these to exclusively occur in individuals who meet criteria for one of these established diagnostic categories. Thus, it is well accepted in the clinics of occupational therapists and pediatricians that there exists a substantial cohort of children who present with significant sensory processing issues and yet do not meet the criteria for ASD or any other “established” neurodevelopmental disorder. These individuals are of major clinical concern, since many of these children suffer substantially, and in the absence of a clearly recognized diagnostic category, their access to services and appropriate treatments is often limited.

Here, we asked whether a cohort of children presenting with an over-responsive SPD phenotype would show deficits in their abilities to integrate audiovisual inputs. A cardinal domain in which audiovisual multisensory integration has a crucial impact on everyday functioning is in speech processing, especially under noisy environmental conditions (MacLeod and Summerfield, 1987; Ross et al., 2007a,b, 2011, 2015; Ma et al., 2009). Therefore, we used a well-established test of multisensory speech-in-noise processing to test the hypothesis that children with SPD would show deficits in their multisensory integrative abilities.

## Materials and Methods

### Participants

Twelve children with a confirmed diagnosis of SPD (nine males, three females, average age = 8.69 years, standard deviation = 2.69) participated in this study. Twelve age-, sex- and IQ-matched typically developing (TD) children served as a control cohort (nine males, three females, average age = 8.06 years, standard deviation = 2.66). Both groups were well matched in terms of intelligence quotients as assessed using the Wechsler Abbreviated Scales of Intelligence (WASI or WASI-2). Average full-scale IQ for the TD group was 104.7 (SEM = 2.77) and for the SPD group was 101.5 (SEM = 2.77), which did not differ significantly (*p* = 0.428). Average verbal IQ was 106.2 (SEM = 2.59) in the TD group and 103.3 (SEM = 2.59) in the SPD group (*p* = 0.448). Average performance IQ was 103.2 (SEM = 3.45) in the TD group and 98.6 (SEM = 3.45) in the SPD group (*p* = 0.357). All participants were native English speakers. Participants were excluded from this study if they had a history of seizures. All children had a normal or corrected-to-normal vision and audiometric threshold evaluation confirmed that all children had a within-normal-limits hearing.

TD children were excluded if they had a history of psychiatric, educational, attentional or other developmental difficulties as assessed by a history questionnaire and were also excluded if their parents endorsed six or more items of inattention or hyperactivity on a DSM-IV checklist for attention deficit disorder (with and without hyperactivity).

Diagnoses of SPD were obtained by a trained occupational therapist (Author ER). To determine inclusion in the SPD group, scores from both the Sensory Processing Scale (SPS) Assessment Version 2.0 and The Short Sensory Profile (SSP) were used. The occupational therapist administered the SPS to develop Global Clinical Impressions (GCI) based on direct observation of structured behavior. These were used to determine whether each participant demonstrated “Sensory Over-Responsivity” (SOR) in at least one of the visual, tactile, or auditory domains^1^. The SSP questionnaire served to quantify caregivers’ observations of various signs of atypical sensory processing across seven sensory domains. Only three domains were used for inclusion in this study: visual/auditory sensitivity, auditory filtering, and tactile sensitivity. Children included in the SPD group scored in the “Definite Difference” range, indicating a score at least two standard deviations from normed means, in at least one of these three domains and in the overall category that draws on all seven domains. Table 1 provides relevant demographic information.

**Table 1:** Sample demographics.

	TD	SPD
*n*	12	12
Age (S.D.)	8.06 (2.66)	8.69 (2.69)
Gender (M/F)	9/3	9/3
FIQ (SE)	104.7 (2.77)	101.5 (2.77)
VIQ (SE)	106.2 (2.59)	103.3 (2.59)
PIQ (SE)	103.2 (3.45)	98.6 (3.45)

*Notes: TD (“typically developed”) represents the control group. SPD represents the sensory processing disorder. FIQ, full scale IQ; VIQ, verbal IQ; PIQ, performance IQ*.

The parents of all child participants provided written informed consent. All procedures were approved by the institutional review board of the Albert Einstein College of Medicine.

### Stimuli and Task

Stimulus materials consisted of digital recordings of 300 simple monosyllabic words spoken by a female speaker. This set of words was a subset of the stimulus material created for a previous experiment in our laboratory (Ross et al., 2007a) and used in several previous studies (Ross et al., 2011, 2015). These words were taken from the “MRC Psycholinguistic Database” (Coltheart, 1981) and were selected from a well-characterized normed set based on their written-word frequency (Kucera and Francis, 1967). The subset of words for the present experiment is a selection of simple, high-frequency words from a child’s everyday environment and is likely to be in the lexicon of children in the age-range of our sample. The recorded movies were digitally re-mastered so that the length of the movie (1.3 s) and the onset of the acoustic signal were similar across all words. Average voice onset occurred at 520 ms after movie onset (*SD* = 30 ms). The words were presented at approximately 50 dBA FSPL, at seven levels of intelligibility including a condition with no noise (NN) and six conditions with added pink noise at 53, 56, 59, 62 and 65 dB SPL. Noise onset was synchronized with movie onset. The signal-to-noise ratios (SNRs) were therefore NN, −3, −6, −9, −12, –15, −18 dB. These SNRs were chosen to cover a performance range in the auditory-alone condition from 0% recognized words at the lowest SNR to almost perfect recognition performance with no noise. The movies were presented on a monitor (NEC Multisync FE 2111SB) at 80 cm distance from the eyes of the participants. The face of the speaker extended approximately 6.44° of visual angle horizontally and 8.58° vertically (hairline to chin). The words and pink noise were presented over headphones (Sennheiser, model HD 555).

The experiment consisted of three randomly intermixed conditions: In the auditory-alone condition (A) the auditory words were presented in conjunction with a still image of the speakers face; in the audiovisual condition (AV) the auditory words were presented in conjunction with the corresponding video of the speaker articulating the words. Finally, in the visual alone condition (V) only the video of the speaker’s articulations was presented. The word stimuli were presented in a fixed order and the condition (the noise level and whether it was presented as A, V, or AV) was assigned to each word randomly. Stimuli were presented in 15 blocks of 20 words with a total of 300 stimulus presentations. There were 140 stimuli for the A and AV conditions respectively (20 stimuli per condition and intelligibility level) and 20 stimuli for the V condition that was presented without noise.

Participants were instructed to watch the screen and report which word they heard (or saw in the V-alone condition). If a word was not clearly understood, participants were encouraged to make their best guess. An experimenter, seated approximately 1 m distance from the participant at a 90° angle to the participant-screen axis, monitored participant’s adherence to maintaining fixation on the screen. Only responses that exactly matched the presented word were considered correct. Any other response was recorded as incorrect.

### Analyses of Task Performance

We submitted percent correct responses in the A and AV conditions as well as AV-gain respectively to separate repeated-measures analyses of variance (RM-ANOVA) with factors SNR and a between-subjects factor of diagnostic group (TD vs. SPD) and AGE as a covariate. Audiovisual enhancement (or AV-gain) was operationalized here as the difference in performance between the AV and the A-alone condition (AV—A). The NN condition was not included in the test for AV-gain to avoid ceiling effects. A univariate ANOVA with factor group and AGE as a covariate was used to test for differences in speechreading. For all ANOVAs we assured the absence of violations of assumptions of equality of variances and equality of covariance matrices (Box test). Violations of the sphericity assumption of the RM-ANOVA were corrected by adjusting the degrees of freedom with the Greenhouse-Geisser correction method. We expected significant main effects of SNR level, and the group as well as an interaction between condition and SNR level replicating previous findings (Ross et al., 2007a,b, 2011, 2015; Ma et al., 2009; Foxe et al., 2015). Age was specifically included as a covariate in these analyses because of our prior work showing clear age effects on speech-in-noise performance across childhood (Ross et al., 2011). As in Ross et al. (2015), estimated marginal means that adjust for this covariate are illustrated in the resulting figures.

## Results

### Performance Differences Between TD and SPD Children

**Figure 1:**
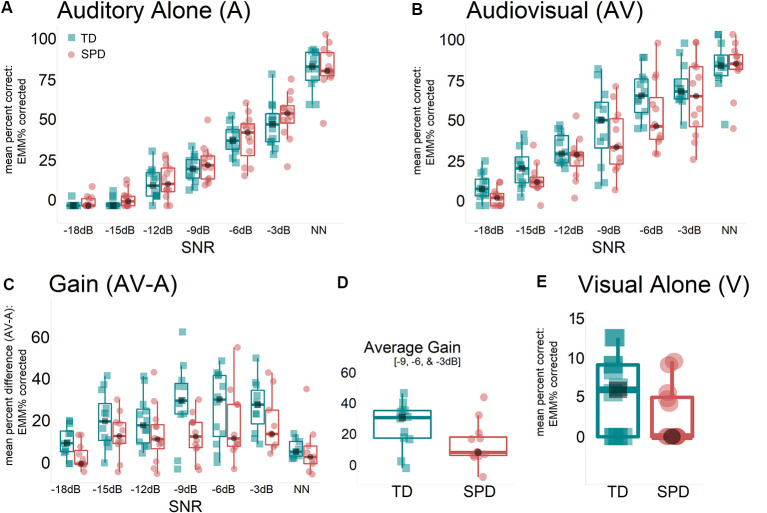
**(A)** Performance in the auditory alone condition does not differ between sensory processing disorder (SPD) and typically developing (TD) children. **(B)** Performance in the audiovisual condition shows a numerical decrease in performance for the SPD children, but this does not reach significance. **(C)** Considering the difference between audiovisual performance and auditory alone performance (i.e., how much multisensory gain is achieved), a clear difference between groups emerges with SPD children showing significantly less gain than is seen in TD children. **(D)** This panel shows the average performance across the three noise levels showing the greatest difference between groups (−9, −6, and −3 dB). The average gain across these three SNR levels is 24.7% in the TD group, compared to 12.6% in the SPD cohort. **(E)** The performance of both TD and SPD children is poor in the visual-alone condition (i.e., lip-reading). There is no significant difference between groups. Note that in all panels estimated marginal means are illustrated, indicating the adjustment in the model for the age covariate.

Performance (% correct) adjusted for the effect of age (marginal means) over SNRs for each group (TD and SPD) and each condition (A, AV) as well as V performance is displayed in Figure 1. The condition with no noise was excluded from the statistical analysis of AV-gain to avoid ceiling effects.

#### Auditory Alone (A)

Similar to our previous studies (Ross et al., 2007a; Foxe et al., 2015), it can be seen that parametric manipulation of SNR influenced speech recognition performance in the A-condition. The RM-ANOVA showed a main effect of SNR (*F*_(4.2,126)_ = 14.23, *p* < 0.001, *η*^2^ = 0.40), which was Greenhouse-Geisser corrected for the violation of sphericity. The factors of SNR and group did not show a significant interaction (*F*_(4.2,126)_ = 0.17, *p* = 0.96, *η*^2^ < 0.01). There was no significant main effect of group (*F*_(1,21)_ = 1.32, *p* = 0.26, *η*^2^ = 0.06), but we found a significant effect of age (*F*_(1,21)_ = 7.78, *p* = 0.01, *η*^2^ = 0.27).

#### Audiovisual (AV)

Here the RM-ANOVA also showed a main effect of SNR (*F*_(3.9,129)_ = 7.12, *p* < 0.001, *η*^2^ = 0.25). Similar to the A-alone RM-ANOVA, this was Greenhouse-Geisser corrected for the violation of sphericity. The factors of SNR and group did not show a significant interaction (*F*_(3.9,129)_ = 0.59, *p* = 0.67, *η*^2^ = 0.03). There was a significant effect of age (*F*_(1,21)_ = 7.76, *p* = 0.01, *η*^2^ = 0.27), but no significant effect of group (*F*_(1,21)_ = 3.12, *p* = 0.09, *η*^2^ = 0.13).

#### Audiovisual Gain (AV-A)

**Figure 2:**
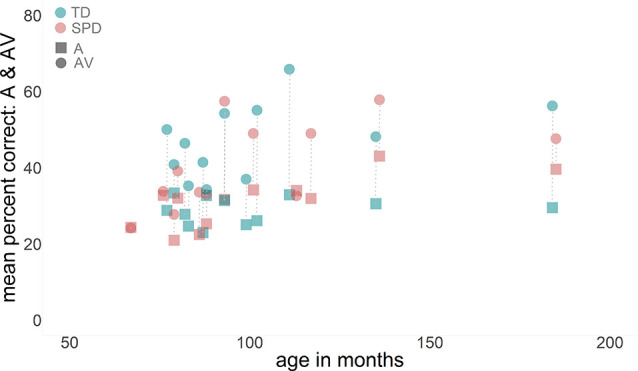
Data are displayed as a function of age (x-axis), with auditory-alone performance represented by square symbols and audiovisual performance represented by the circle symbols. Dotted lines join each participants’ two data points together. There was no significant effect of age on audiovisual gain.

AV-gain was obtained by linearly subtracting A-only response accuracy from AV response accuracy over six SNRs, excluding the NN condition. The RM-ANOVA showed no main effect of SNR (*F*_(3.7,105)_ = 0.39, *p* = 0.8, *η*^2^ = 0.02) when using a Greenhouse-Geisser correction for the violation of sphericity. There was no significant interaction effect between SNR and group (*F*_(3.7,105)_ = 0.23, *p* = 0.91, *η*^2^ = 0.01). Critically, the SPD group showed less AV-gain (*M* = 10.63; *SD* = 14.7) over all six SNRs than the TD group (*M* = 20.9; *SD* = 14.7) which was indexed by a significant main effect of group (*F*_(1,21)_ = 7.11, *p* = 0.01, *η*^2^ = 0.25). Age had no significant effect on AV-gain (*F*_(1,21)_ = 2.33, *p* = 0.14, *η*^2^ = 0.10). An additional paired samples *t*-test was carried out comparing AV (*M* = 33.61; *SD* = 11.64) with A means (*M* = 22.76; *SD* = 6.5) excluding the NN condition within the SPD group. The significant *t-statistic* confirmed that significant AV- gain was achieved by this group despite the sizable differences to the TD group *t*_(11)_ = −4.29, *p* = 0.001. Figure 2 displays the AV-gain data as a function of age for completeness in reporting.

#### Visual Only (V)

A Univariate Analysis of Variance with the factor group, age as a covariate and the V condition as a dependent variable was performed to assess group differences in the speechreading. The F-test did not return a statistical difference between SPD (*M* = 2.76; *SD* = 3.73) and TD children (*M* = 5.28; *SD* = 5.07; *F*_(1,21)_ = 1.85, *p* = 0.19, *η*^2^ = 0.08).

## Discussion

It has long been speculated that multisensory integration deficits might lie at the core of the sensory processing anomalies observed in children who show hyper- or hypo-sensitivities to everyday sensory inputs. Here, we tested the abilities of children with a hyper-responsive SPD phenotype to recognize speech inputs under varying levels of background noise using a well-established assay of multisensory speech integration. It is clear from decades of work that neurotypical individuals gain substantial benefits in speech comprehension from both seeing and hearing a speaker under such circumstances (Sumby and Pollack, 1954; Erber, 1969), so assays of multisensory speech integration have become one of the primary means by which multisensory processing abilities are measured in various clinical and neurotypical groups (Smith and Bennetto, 2007; Irwin et al., 2011; Hahn et al., 2014; Foxe et al., 2015; Cuppini et al., 2017; Beker et al., 2018). The current results reveal a significant deficit in the abilities of children with an SPD to benefit from multisensory speech inputs, relative to a cohort of matched typically developing control participants.

It is worth pointing out that the age-range of the current SPD cohort is relatively young, with an average age of 8.7 years. This is important because, in previous work in children with ASD, we showed that multisensory speech deficits were particularly prominent in this age-range, but that they appeared to resolve in children after about the age of 13 years (Foxe et al., 2015). It will be of considerable interest to see if the same general delayed developmental trajectory for multisensory processing that we observed in ASD children can also be observed in SPD children, so a study in a cohort of teenagers and young adults is merited. Similarly, we have shown multisensory processing deficits for much more fundamental stimuli than speech (i.e., simple tones and visual flashes) in ASD, which points to a more general multisensory processing deficit in that population. In a partner study to the current investigation of speech integration, we also assessed response speeds to very basic audiovisual inputs relative to unisensory inputs (Molholm et al., 2020). When neurotypical children and adults are asked to respond in this fashion, it is typical to observe a significantly speeded up response to bisensory audiovisual inputs relative to unisensory (i.e., auditory-alone or visual-alone inputs; Molholm et al., 2002; Mégevand et al., 2013), although this speeding is relatively modest in children in the age-range of the current study (Brandwein et al., 2011). Nonetheless, when children with an SPD were compared to TD children for this multisensory response speeding, we found that they did not show the typical response speeding. Descriptive comparison with Brandwein et al. (2013) suggests that they show a similar response pattern to that seen in children with ASD on this behavioral metric (Brandwein et al., 2013). Thus, taken together, these two studies on SPD suggest multisensory integration deficits for both basic audiovisual and higher-order social stimuli, at least at the behavioral level, and highlight the fact that these multisensory deficits are quite similar to those observed in ASD.

Returning to the age-range of the current cohort, it bears pointing out that in prior work where we mapped the developmental trajectory of multisensory speech integration across childhood (see Figure 2 in Ross et al., 2011), the audiovisual gain was quite immature in children in the age-range under study here. In adults and older children, a highly characteristic “tuning” pattern is seen for audiovisual enhancement of speech recognition, with a distinct peak seen at the −12 dB signal-to-noise ratio. However, in the Ross study of 2011, no such peak was seen in younger children (aged 5–7 years), and this pattern only began to emerge in 10–12-year-olds, and even then, it was considerably attenuated relative to adults. In the current cohorts, the average age was 8.5 years, with only two children in each group above 10 years. Figure 1C shows wholly similar audiovisual gain patterns in the current cohort to those seen in the youngest group of Ross et al. (2011), with maximal gain seen at the noise levels between −3 dB and −9 dB, reaching an average of 24.7% gain across these three noise levels in the control group. This compares with an average gain of just 12.6% across these same noise levels in the SPD cohort. It is instructive to consider this against our prior adult data, where the maximal gain is in the region of 50% at −12 dB.

There have been prior efforts to characterize multisensory integration processes in SPD children. For example, multisensory integration of auditory and somatosensory inputs (passively observed) was investigated in a cohort of 20 sensory over-responsive children using event-related potentials (ERPs; Brett-Green et al., 2010). The authors showed multisensory integration effects at multiple time points during sensory processing, so it was clear from the results that at least some aspects of integrative processing were intact (as in our partner article Molholm et al., 2020; this volume), but in that study, there was no comparison control group, so direct inferences about aberrant processing could not be made. Nonetheless, the authors did note some differences in the integration effects they observed relative to prior reports in the literature (Foxe et al., 2000).

There is also evidence from ERP assays for sensory gating abnormalities in the auditory modality (Davies and Gavin, 2007; Davies et al., 2009). In this pair of articles, auditory click pairs were presented in quick succession (500 ms inter-click-interval), and as is typically done in such studies, the amplitude of the ERP to the second click was compared to that of the first click. In the TD control group, a clear decrease in the amplitude of the response to the second stimulus of the pair, relative to the first, is usually observed. Davies and Gavin found that this “adaptation” was somewhat attenuated in SPD. Interestingly, the adaptation effect was found to mature with age in the TD population whereas this association was not as evident in the SPD cohort. A comprehensive investigation of adaptation across the three major sensory systems and also between sensory systems would be of considerable interest in SPD (Andrade et al., 2015, 2016; Uppal et al., 2016). It is rather intuitive that a decrement in the ability to gate repetitive (unimportant/obtrusive) stimulation streams could well be a significant contributor to the SPD phenotype, but considerable additional work will be required to establish whether this is, in fact, consistently observed in this population.

Another finding of potential note in the current study is to be found in the unisensory auditory data, where the children with SPD, perhaps surprisingly, showed no detectable deficits in their abilities to recognize words across the various noise levels when they were presented during the auditory-alone condition. Given the sensory defensive phenotype associated with this population, it might well have been expected that higher background noise conditions would have selectively impacted their performance. Instead, all effects appear to be focused on the multisensory condition. Here again, this finding largely parallels the pattern that we previously observed in children with ASD in which only small differences were found in the auditory condition (Foxe et al., 2015), another population in which there has been much theorizing about susceptibility to external noise conditions (Kanakri et al., 2017; Park et al., 2017). The current data, therefore, suggest that susceptibility to external auditory noise, while it may be uncomfortable for these individuals, something we did not measure explicitly here, does not necessarily impact their sensory-perceptual abilities. Of course, only a limited range of external noise conditions was employed here, and at its loudest, the pink noise-masking was titrated to approximately 65 dB SPL, which is not a particularly uncomfortable listening level. The fact that children were presented with 300 stimulus presentations may also have resulted in a measure of successful habituation to the various noise levels. It will fall to future work to determine whether more uncomfortable background noise levels would also reveal unisensory word recognition deficits in SPD.

It is also of interest to those in the multisensory integration field that the current data do not accord with the so-called “inverse effectiveness” principle. That is, one of the key observations from early single-unit electrophysiology work in animal models was that the magnitude of multisensory response enhancements occurred when the constituent unisensory inputs were minimally effective in evoking responses (Wallace et al., 1996). The operation of this principle is also seen in human electrophysiological studies when the task of the participant is simply to orient to, or to detect, a multisensory stimulus input (Senkowski et al., 2011). However, it has repeatedly been shown that this principle does not apply well to speech recognition data, and in earlier work, we posited that the speech integration system was likely tuned for intermediate signal-to-noise ratios (Ross et al., 2007a). In subsequent modeling work, we showed that Bayesian estimates of optimal multisensory speech integration, given the inherent high dimensionality of the semantic feature space, predicted precisely this intermediate pattern of results (Ma et al., 2009).

## Study Limitations

The main limitation of this study is the relatively modest SPD cohort size (*N* = 12) and relatedly, that we were not in a position to assess multisensory integration across a greater span of ages to establish whether the developmental trajectory of this capacity differs in this population. It should also be pointed out that the use of pink noise as an experimental proxy for background environmental noise is not a fully realistic recapitulation of the sorts of noise environments under which individuals are usually required to extract speech from noise, and that future work using more real-world conditions is certainly merited. It will also be of significant interest to understand the role of attention in speech integration processes in future work (Senkowski et al., 2008; O’Sullivan et al., 2019).

## Conclusion

For a sizable minority of children, simple sensory processing of everyday inputs can prove an overwhelming challenge (Miller et al., 2007, 2009). While such sensory phenotypes are recognized as highly prevalent in neurodevelopmental disorders such as Autism, many of those suffering from an SPD find it difficult to receive appropriate clinical care. Here, we show that school-aged children with an SPD show a deficit in the ability to benefit from the combination of both seen and heard speech inputs under noisy environmental conditions, suggesting that these children do not benefit from multisensory integrative processing to the same extent as their typically developing peers. The deficit is highly similar to multisensory speech processing deficits previously described in similarly aged children with ASD, perhaps pointing to a common endophenotypic source. In light of parallel work showing a deficit in simple response speeding to basic audiovisual inputs in children with SPD, emerging evidence suggests that there may be a general sensory integration deficit in these children, in line with one of the major theories in this domain.

## Data Availability Statement

The dataset supporting the conclusions of this article will be made available in the figshare repository https://figshare.com/.

## Ethics Statement

This study was reviewed and approved by the institutional review board of The Albert Einstein College of Medicine (Protocol Reference Number #2011-210). Written informed consent was obtained from parents or legal guardians, where possible assent from the patient was also ascertained, and all aspects of the research conformed to the tenets of the Declaration of Helsinki.

## Author Contributions

JF and LR designed and implemented this study. The technical team at the CNL collected the bulk of the data. ER recruited and phenotyped the patients. VD performed the main data analyses and produced the initial data illustrations. AF contributed to the final data illustration. JF, SM, LR, and VD discussed and conducted statistical analyses. JF wrote the first draft of the article and received extensive editorial input on subsequent drafts from all of the co-authors. All co-authors have evaluated and approved the final version of this article, and all co-authors had full and unfettered access to the datasets used to generate this report. All authors read and approved the final manuscript.

## Conflict of Interest

The authors declare that the research was conducted in the absence of any commercial or financial relationships that could be construed as a potential conflict of interest.

## Footnotes

^1^The SPS assesses seven domains of sensory processing for three different types of abnormality, but for the purposes of this study, only SOR in three chosen domains factored into classification.
